# Elevated CO_2_ has concurrent effects on leaf and grain metabolism but minimal effects on yield in wheat

**DOI:** 10.1093/jxb/eraa330

**Published:** 2020-07-20

**Authors:** Guillaume Tcherkez, Sinda Ben Mariem, Luis Larraya, Jose M García-Mina, Angel M Zamarreño, Alberto Paradela, Jing Cui, Franz-Werner Badeck, Diego Meza, Fulvia Rizza, James Bunce, Xue Han, Sabine Tausz-Posch, Luigi Cattivelli, Andreas Fangmeier, Iker Aranjuelo

**Affiliations:** 1 Research School of Biology, ANU Joint College of Sciences, Australian National University, Canberra Australia; 2 Institut de Recherche en Horticulture et Semences, INRA d’Angers, Université d’Angers, Structure Fédérative de Recherche QUASAV, Beaucouzé, France; 3 AgroBiotechnology Institute (IdAB), CSIC-Government of Navarre, Mutilva, Spain; 4 Institute for Multidisciplinary Applied Biology, Departamento de Agronomía, Biotecnología y Alimentación, Universidad Pública de Navarra, Pamplona, Spain; 5 Facultades de Ciencias y Farmacia y Nutrición, Grupo de Biología y Química Agrícola (Departamento de Biología Ambiental), Universidad de Navarra, Pamplona, Spain; 6 Centro Nacional de Biotecnología-CSIC, Madrid, Spain; 7 CREA Research Centre for Genomics and Bioinformatics, Fiorenzuola d’Arda, Italy; 8 Institute of Landscape and Plant Ecology, University of Hohenheim, Stuttgart, Germany; 9 Adaptive Cropping Systems Lab, Beltsville Agricultural Research Center, Agricultural Research Service, US Department of Agriculture, Beltsville, MD, USA; 10 Institute of Environment and sustainable Development in Agriculture, Chinese Academy of Agricultural Sciences (IEDA, CAAS), Beijing, China; 11 Department of Agriculture, Science and the Environment, School of Health, Medical and Applied Sciences, CQUniversity Australia, Kawana, QLD, Australia; 12 Australian National University

**Keywords:** Climate change, ree-air CO_2_ enrichment (FACE), multiple locations, N/C metabolism, physiology, varieties, wheat

## Abstract

While the general effect of CO_2_ enrichment on photosynthesis, stomatal conductance, N content, and yield has been documented, there is still some uncertainty as to whether there are interactive effects between CO_2_ enrichment and other factors, such as temperature, geographical location, water availability, and cultivar. In addition, the metabolic coordination between leaves and grains, which is crucial for crop responsiveness to elevated CO_2_, has never been examined closely. Here, we address these two aspects by multi-level analyses of data from several free-air CO_2_ enrichment experiments conducted in five different countries. There was little effect of elevated CO_2_ on yield (except in the USA), likely due to photosynthetic capacity acclimation, as reflected by protein profiles. In addition, there was a significant decrease in leaf amino acids (threonine) and macroelements (e.g. K) at elevated CO_2_, while other elements, such as Mg or S, increased. Despite the non-significant effect of CO_2_ enrichment on yield, grains appeared to be significantly depleted in N (as expected), but also in threonine, the S-containing amino acid methionine, and Mg. Overall, our results suggest a strong detrimental effect of CO_2_ enrichment on nutrient availability and remobilization from leaves to grains.

## Teaser

A study of wheat grown at five FACE facilities around the world shows a low effect of CO_2_ on grain yield, and reveals relevant modifications in N and Mg metabolism.

## Introduction

Wheat represents 27% of global grain production (FAOSTAT, 2018: http://www.fao.org/faostat/en/#home) and, along with corn and rice, is a major component of the human diet because of its high nutritional value: it is a source of carbohydrates, macroelements and micro (trace) elements, protein, and free amino acids. Bread wheat (*Triticum aestivum* L.) constitutes up to 95% of global wheat production, while durum wheat (*Triticum turgidum* ssp. *durum*) makes up the remainder (He *et al.*, 2013). While bread wheat is cultivated in all parts of the world (Reynolds *et al.*, 2012), durum wheat is mostly grown in the Mediterranean region (Oliveira *et al.*, 2012). Like other crops, wheat cultivation will face serious challenges in the coming decades, with a need to increase production despite challenging environmental conditions caused by climate change. Doubling production by 2050 to meet the anticipated demand is expected to be difficult, since yield would have to increase by 2.4% per year globally whereas the actual rate of increase is only 1.3% per year and, furthermore, ~39% of wheat-cultivating areas have shown no increase in yield in a decade (Ray *et al.*, 2012). Among important questions that must be answered to understand how wheat yield responds to changing climatic conditions is the potential impact of rising atmospheric CO_2_.

Atmospheric CO_2_ has progressively increased since the beginning of the industrial revolution, and the CO_2_ mole fraction is anticipated to double (the predicted mole fraction ranges between 730 and 1020 ppm) by 2100 (Meehl *et al.*, 2007). Since photosynthetic activity in C_3_ plants is CO_2_-limited, it was predicted that the increase in atmospheric CO_2_ would stimulate photosynthesis and thus plant growth (Bowes, 1993). However, crop response to elevated CO_2_ varies widely, depending on growth (cultivation) conditions and inter- and intra-specific variability. While a CO_2_-driven increase in yield is generally observed, there is substantial variation, with sometimes no yield gain at all (Ainsworth and Long, 2005; Högy and Fangmeier, 2008). This effect mostly comes from photosynthetic acclimation to high CO_2_, which in turn disfavours grain production. In wheat, cultivation under free-air atmospheric CO_2_ enrichment (FACE) leads, on average, to a yield enhancement of only 10% (Kimball, 2010, 2016). Photosynthesis acclimates to elevated CO_2_ via lower average stomatal conductance (*g*_c_), lower photosynthetic capacity (maximum carboxylation rate *V*_cmax_), and/or lower ribulose 1,5-bisphosphate regeneration ability (Miglietta *et al.*, 1996; Adam *et al.*, 2000; Rogers and Humphries, 2000; Wechsung *et al.*, 2000; Zhang *et al.*, 2009). Of course, wheat response to elevated CO_2_ also depends on water and N supply. In general, elevated CO_2_ leads to a higher relative increase in wheat grain yield under water-restricted conditions (up to 20%) compared with rain-fed plants (up to 10%), although yield absolute values are, as expected, lower under water restriction (Kimball *et al.*, 1995; Hunsaker *et al.*, 1996; Tubiello *et al.*, 1999). In addition, the extent to which yield is stimulated by elevated CO_2_ under low water input depends on the sowing date and whether additional irrigation is implemented under control (well-watered) conditions (O’Leary *et al.*, 2015). Unsurprisingly, minimal effects of elevated CO_2_ on photosynthesis and yield are observed under N-limited conditions (Li *et al.*, 2000; Grant *et al.*, 2001; Kimball *et al.*, 2001a; Pacholski *et al.*, 2015; Walker *et al.*, 2017; Manderscheid *et al.*, 2018). Furthermore, elevated CO_2_ triggers developmental changes that are detrimental to yield. In particular, wheat cultivated under FACE has a lower shoot-to-root ratio (Wechsung *et al.*, 1995; Pacholski *et al.*, 2015) and perhaps an increased root exudation rate (thereby increasing organic matter deposition in soil and thus soil respiration) (Pendall *et al.*, 2001) and accelerated flag leaf senescence (Zhu *et al.*, 2009). By contrast, elevated CO_2_ causes an increase in tiller number (Yang *et al.*, 2007a, b) and has a marginal effect on transpiration, making plants slightly more water-efficient, especially under high N supply (Hunsaker *et al.*, 1996; Erbs *et al.*, 2009; O’Leary *et al.*, 2015; Kimball, 2016; Manderscheid *et al.*, 2018).

In addition to such quantitative changes in crop production and photosynthesis, elevated CO_2_ leads to modifications in leaf biochemical composition and grain quality. Under elevated CO_2_, wheat leaves invest more C in cellulose and flavonoids (Akin *et al.*, 1995; Peñuelas *et al.*, 2000) and are generally less N-rich, with a higher C/N ratio (Zhu *et al.*, 2009; Aranjuelo *et al.*, 2015) and lower amounts of proteins (or transcripts encoding them) involved in photosynthesis (Calvin cycle enzymes) (Nie *et al.*, 1995; Adam *et al.*, 2000; Zhang *et al.*, 2009; Pandey *et al.*, 2017). Proteomics conducted on leaves sampled during anthesis in wheat cultivated in a CO_2_-enriched greenhouse (at 700 µmol mol^−1^ CO_2_) have shown that the Rubisco content did not increase, while there was a slightly higher content of Rubisco activase (Aranjuelo *et al.*, 2015). When grown under FACE, leaves have been found to contain less Rubisco and more mitochondrial malate dehydrogenase (NAD-dependent), suggesting a change in respiratory metabolism (respiratory CO_2_ loss) (Pandey *et al.*, 2017). The detrimental effect of CO_2_ on the photosynthetic machinery of flag leaves is accompanied by an increase of ear photosynthesis and a faster decline in flag leaf N due to earlier senescence (Sicher and Bunce, 1998; Zhu *et al.*, 2009). Taken as a whole, elevated CO_2_ has a major effect on leaf N metabolism, which in turn impacts on remobilization and thus grain filling during maturation.

Unsurprisingly therefore, wheat grains (and flour produced from them) produced at elevated CO_2_ have a generally lower nutritional quality, with lower N (and S) content, less protein, and more starch and fibres (Kimball et al., 2001a, b; Högy *et al.*, 2013; Wroblewitz *et al.*, 2013). We have recently shown that when wheat plants are grown in the greenhouse at elevated CO_2_ under ample N supply, grains are less N-rich and do not have the same kinetics of gliadin and glutenin accumulation (Soba *et al.*, 2019). In this case, it has been suggested that this effect did not come from deficient N remobilization in leaves but rather a N dilution effect, whereby the quantity (or size) of grains increased while the total available N for remobilization did not change. In addition, under FACE conditions, an increase in the glutenin-to-gliadin ratio has been found (Pandey *et al.*, 2017), as well as a general decrease in all storage proteins except for globulins and albumins (Wieser *et al.*, 2008, but see Verrillo *et al.*, 2017, where globulins also decreased). Metabolic analyses have demonstrated that at elevated CO_2_, grains contain more secondary metabolites (shikimate and quinate) and free hexoses (fructose and glucose) but less sucrose and alanine, regardless of developmental stage. At maturity, grains are generally depleted in free amino acids, except for the branched-chain amino acids valine, leucine, and isoleucine, which are significantly more abundant (Wroblewitz *et al.*, 2013; Soba *et al.*, 2019). Furthermore, it has been suggested that the metabolism of lysine recycling changes and favours the saccharopine pathway over pipecolate (Soba *et al.*, 2019). It is also worth noting that elevated CO_2_ causes a decline in the content of several microelements in wheat grains, such as Fe and Zn (Wroblewitz *et al.*, 2013; Pandey *et al.*, 2017; Beleggia *et al.*, 2018), further impacting on nutritional quality.

Nevertheless, the effect of elevated CO_2_ likely depends not only on the cultivation conditions (water and N supply, time of sowing, and temperature), but also on soil properties and the species and cultivar of wheat. For example, wheat lines of different ploidy do not respond similarly to elevated CO_2_ in FACE experiments, with hexaploid bread wheat being the most responsive in terms of yield (but the least in terms of photosynthesis) (Uprety *et al.*, 2009). There is also substantial variability between cultivars, from no response at all to significant response to CO_2_, with little correlation between yield and photosynthesis, but significant correlation with N content and specific leaf area (Thilakarathne et al., 2013, 2015) or transpiration efficiency (Tausz-Posch *et al.*, 2013b; but see also Bourgault *et al.*, 2013). The impact of elevated CO_2_ on both yield and elemental contents has been found to depend on the cultivar (Fernando *et al.*, 2014; Fares *et al.*, 2016; Houshmandfar *et al.*, 2016; Beleggia *et al.*, 2018). Variability in the response to elevated CO_2_ between wheat cultivars likely results from differences in N and chlorophyll content, transpiration efficiency, or tillering capacity (Tausz-Posch *et al.*, 2013a; Veres *et al.*, 2017). Modelling has also suggested that variation in the response of wheat lines to elevated CO_2_ can result from differences in development and allocation (sink/source relationships) (Wolf *et al.*, 2002). In addition, it is probable that the response to elevated CO_2_ may vary when soil properties change. This aspect is less documented but, in principle, the spatial variability of wheat yield in the field under ambient CO_2_ has been shown to be related to soil P or organic C content, soil structure, or the proportion of carbonates (Miller *et al.*, 1988; Bhatti *et al.*, 1991; Moulin *et al.*, 1994). Since in wheat there is a relationship between root deposition stimulated by elevated CO_2_, root-driven acidification, and cation mobility (Cheng *et al.*, 2010), the effect of elevated CO_2_ could be modulated by soil Na, Ca, and K content and pH.

Taken as a whole, whenever there are differences among growth conditions and cultivars, it is difficult to understand the physiological mechanisms underlying the response to elevated CO_2_. In fact, it could well be that FACE experiments conducted separately in different countries with different cultivars are not comparable, not only because soil and climate conditions vary but also because the response to CO_2_ is cultivar-specific. To tackle this issue, we conducted a targeted metabolic analysis of samples collected during FACE experiments (at 550 µmol mol^−1^ CO_2_) conducted in different countries (USA, Australia, Germany, China, and Italy) with different species (*T. aestivum* and *T. turgidum* ssp. *durum*) and cultivars (Table 1; Supplementary Fig. S1). We carried out elemental analyses (macroelements and some microelements), quantitation of amino acids and sugars, and targeted protein analysis in leaves and grains. Our objective was to assess whether a significant effect of elevated CO_2_ could be observed in both leaves and grains across countries and wheat lines and, by doing so, to look at the potential effect of leaf-to-grain nutrient remobilization on CO_2_ responsiveness. Our data could also be exploited further by exploring the potential relationship between yield (treated as a quantitative response variable) and grain biochemical composition. Our working hypothesis was that (i) detrimental effects of elevated CO_2_ on photosynthesis and N assimilation were associated with significant changes in protein and amino acid composition in leaves, and thus (ii) the amino acid composition of grains was affected, reflecting alteration of both the provision and recycling of nitrogenous compounds; and (iii) there might be a relationship between grain metabolic features and yield.

## Materials and methods

### Plant material and experimental design

The experiment was conducted with durum wheat (cultivars Miradoux, Duramant, Claudio, and Simeto) and bread wheat (cultivars Janz, Kite, Norin, Triumph, Milan, and PRL). The study involved five FACE facilities located in Stuttgart-Hohenheim (Germany), Fiorenzuola (Italy), Changping-Beijing (China), Horsham (Australia), and Beltsville (USA), in which plants were exposed to ambient (400 ppm) and elevated (550 ppm) CO_2_ conditions. FACE facilities included three to five rings (for each CO_2_ concentration condition) with a diameter of 12–14 m each or (in Stuttgart-Hohenheim) 2×2 m square plots. In all cases, CO_2_ enrichment was performed from sunrise to sunset (in Australia and Italy) or 24 h a day (in China, Germany, and the USA) and throughout the entire growing season. The principle of operation and the performance of the FACE systems used here have been described previously (Mollah *et al.*, 2009; Fangmeier *et al.*, 2016; Fares *et al.*, 2016). When plants reached the stem elongation (Z31) and anthesis half-way (Z65) stages (BBCH), sampling and gas-exchange analyses were carried out in the last expanded leaves (Z31) or flag leaves (Z65). Collected leaf samples were immediately frozen in liquid N_2_ and stored at –80 °C for later analyses. Finally, at maturity stage (Z90), grain samples were collected for agronomic and metabolic analyses.

### Gas exchange determinations

Analyses were carried out in healthy expanded flag leaves grown under ambient or elevated CO_2_ conditions. The light-saturated rate of CO_2_ assimilation (*A*_sat_), stomatal conductance (*g*_s_), and intercellular CO_2_ concentration (*c*_i_) were estimated at a photosynthetic photon flux density of 1500–1600 µmol m^−2^ s^−1^ using equations developed by von Caemmerer and Farquhar (1981). The vapour pressure deficit was 1.5 kPa. CO_2_ response curves were constructed by a series of measurements whereby photosynthesis was determined first under standard conditions (400 µmol mol^−1^), then at low CO_2_, then back to standard conditions, and then at high CO_2_: overall, the sequence of CO_2_ concentrations in the reference channel was 400, 200, 100, 50, 200, 400, 600, 750, and 950 µmol mol^−1^. Estimations of the maximum carboxylation velocity of Rubisco (*V_c_*_max_) and the maximum electron transport rate contributing to ribulose 1,5-bisphosphate regeneration (*J*_max_) (measurements made in Germany) were made using the method of Harley *et al.* (1992).

### Mineral composition

C and N concentration (%) analyses were determined using an elemental analyzer (EA, Carlo Erba Strumentazione, Milan, Italy). Micronutrient and macronutrient concentrations were determined by inductively coupled plasma/optical emission spectrometry (ICP/OES, iCAP 6500 Duo, Thermo Fisher Scientific, Waltham, MA, USA).

### C isotope discrimination

Carbon isotope composition was determined using an elemental analyzer (EA1108; Carlo Erba Strumentazione, Milan, Italy) coupled to an isotope ratio mass spectrometer (Delta C; Finnigan MAT, Bremen, Germany). Values were expressed as delta values δ ^13^C=(R_sample_/R_standard_)–1 and expressed in ‰, where R_standard_ is the ^13^C-to-^12^C ratio of the international standard (V-PDB). The carbon isotope discrimination (Δ) was calculated as follows:

$$\Delta \left(0/00\right)=\left(\delta^{\text{13}}{\text{C}}_{\text{air}}-\delta^{\text{13}}{\text{C}}_{\text{sample}}\right)/\left(\text{1}+\delta^{\text{13}}{\text{C}}_{\text{sample}}\right)$$ (1)

where δ ^13^C_air_ (‰) is the isotope ratio in atmospheric CO_2_. The addition of (industrial) CO_2_ in the course of the FACE experiments was such that δ ^13^C_air_ was lower than the natural ^13^C abundance in the atmosphere (near –8‰). The δ ^13^C value of the added CO_2_ was known (for the experiment in Germany) or calculated from mass balance, taking advantage of the known contribution of added CO_2_ to total CO_2_: *c*=(550–400)/550=27.2%. In fact, if the isotope fractionation under ambient CO_2_ and under FACE conditions was the same, then the proportion of C coming from the added CO_2_ could be calculated as *c*=(δ _OM,amb_–δ* _OM,FACE_)/(δ _CO2,amb_–δ _CO2,added_), where OM refers to organic matter and amb refers to ambient conditions, and δ ^13^C values are abbreviated to δ to simplify the notation. δ* _OM,FACE_ is the δ ^13^C value of organic matter under FACE conditions if the fractionation had not changed compared with ambient conditions. In practice, the isotope fractionation could have varied, and thus (neglecting denominators): δ* _OM,FACE_ =δ _OM,FACE_+Δ _FACE_–Δ _amb_, where Δ _FACE_ and Δ _amb_ are the net photosynthetic fractionation under FACE and ambient conditions, respectively. These equations can be combined to give *c*=(δ _OM,amb_–δ _OM,FACE_)/(δ _CO2,amb_–δ _CO2,added_)–(Δ _FACE_–Δ _amb_)/(δ _CO2,amb_–δ _CO2,added_). The right term is relatively small since added industrial CO_2_ was naturally ^13^C depleted and thus δ _CO2,amb_–δ _CO2,added_ is much larger than Δ _FACE_–Δ _amb_. That is, by neglecting the right term, δ _CO2,added_ can be estimated (and therefore Δ _FACE_ with equation 1) by imposing *c*=0.272. Note that such an approximation was not critical, since even under the assumption (Δ _FACE_–Δ _amb_)/(δ _CO2,amb_–δ _CO2,added_) represented 0.10 (i.e. 40% error in *c*); this would ultimately cause an error of only 1‰ in Δ _FACE_.

### Abscisic acid content

Extraction, purification, and quantification of abscisic acid (ABA) were carried out as described by Torres *et al.* (2018), using a high-resolution mass spectrometry (HPLC-ESI-HRMS) system, with some modifications: freeze-dried material (15 mg) was used instead of frozen powdered material (0.1 g), and the residue obtained after final evaporation (SpeedVac) was redissolved in 0.25 ml methanol instead of 0.5 ml.

### Soluble sugar and starch content

Sucrose, glucose, and fructose content were determined using a Beckman P/ACE5500 capillary electrophoresis system (Beckman Instruments, Fullerton, CA, USA), following the method of Cabrerizo *et al.* (2001). Starch content in the pellet was determined according to Ethier and Livingston (2004).

### Amino acid content

Amino acids were derivatized at 22–25 °C for 12–16 h with 1 mM fluorescein isothiocyanate dissolved in 20 mM acetone/borate, pH 10. The content of single amino acids was determined by capillary electrophoresis in a Beckman-Coulter PA-800 system.

### Protein content

Samples were previously quantified by microBCA analysis (Pierce) and similar amounts (5 µg per sample) were individually dissolved in 8 M urea, 25 mM ammonium bicarbonate, reduced with DTT, and alkylated with iodoacetamide, according to a method described by López-Ferrer *et al.* (2004). Digested samples were diluted with 0.2% trifluoroacetic acid in water and subjected to multiple reaction monitoring analysis using a 1D Plus nanoLC Ultra system (Eksigent, Dublin, CA, USA) interfaced to a Sciex 5500 QTRAP triple quadrupole mass spectrometer (Sciex, Framingham, MA, USA) equipped with a nano-electrospray ionization source and controlled by Analyst v.1.5.2. software (ABSciex). Trypsin-digested samples were loaded online on a C18 PepMap 300 µm internal diameter × 5 mm trapping column (5 µm, 100 Å, Thermo Scientific) and separated using a BioSphere C18 75 µm internal diameter × 150 mm capillary column (3 µm, 120 Å, Nanoseparations). A list of 84 transitions (usually 3–4 per peptide, with a preference toward higher-mass y series ions), corresponding to 21 unique peptides selected for 10 different proteins, was monitored. Skyline software determined automatically the collision energy values for the candidate peptides according to MacLean *et al.* (2010).

### Statistical analysis

To explore the effect of CO_2_, univariate statistics were conducted with a two-way ANOVA, with one factor representing ‘conditions’ (time of sampling, country, and species/cultivar) while the second factor was CO_2_. It was not possible to carry out a three-way analysis because not all species/cultivars were represented in all countries. Thus, here, the two-way ANOVA was constructed with the factor ‘conditions’ having 12 possible qualitative values (combinations time–country–cultivar) and the factor ‘CO_2_’ having two values (ambient or elevated). Unless otherwise stated, statistical significance was accepted when *P*<0.05. Multivariate statistics were carried out using orthogonal projection on latent structure (OPLS; with Simca®, Umetrics), using CO_2_ as the predicted Y variable (while ‘conditions’ are embedded into orthogonal dimensions), and metabolic features (elemental contents, metabolites, and proteins) as predicting X variables. Before running the OPLS, a principal component analysis was conducted to check the presence of outliers (samples outside the Hotelling’s ellipse). The performance of the OPLS was assessed using the correlation coefficient between predicted and observed Y (*R*^2^), the cross-validated correlation coefficient (*Q*^2^), the *Q*^2^ intercept of the permutation test (which was checked to be negative), and the *P*-value of testing the OPLS model against a random-error model (i.e. average ±error) via a χ ^2^ test (this *P*-value is referred to as *P*_CV-ANOVA_). Univariate and multivariate analyses were combined using –log(*P*-value) (univariate) plotted against the OPLS loading (p_corr_) in a volcano plot. To explore the relationship between yield and grain composition, we conducted (i) an OPLS using yield as the predicted Y variable (with the same parameters of performance as described above) and (ii) a univariate analysis by linear regression (done in R). Linear regression was done without and with variable elimination. Without variable elimination, each variable was correlated with yield separately and the associated *P*-value is reported. To carry out variable elimination, we used a multiple linear regression. It was carried out via sampling subsets (regsubsets) of eight variables (304 iterations) and a correlation plot was generated to select the eight most correlated variables overall. Then, a multiple linear regression model (lm) comprising only these eight variables was generated and the *P*-value for each variable was calculated.

## Results

### Photosynthetic parameters

**Fig. 1.**
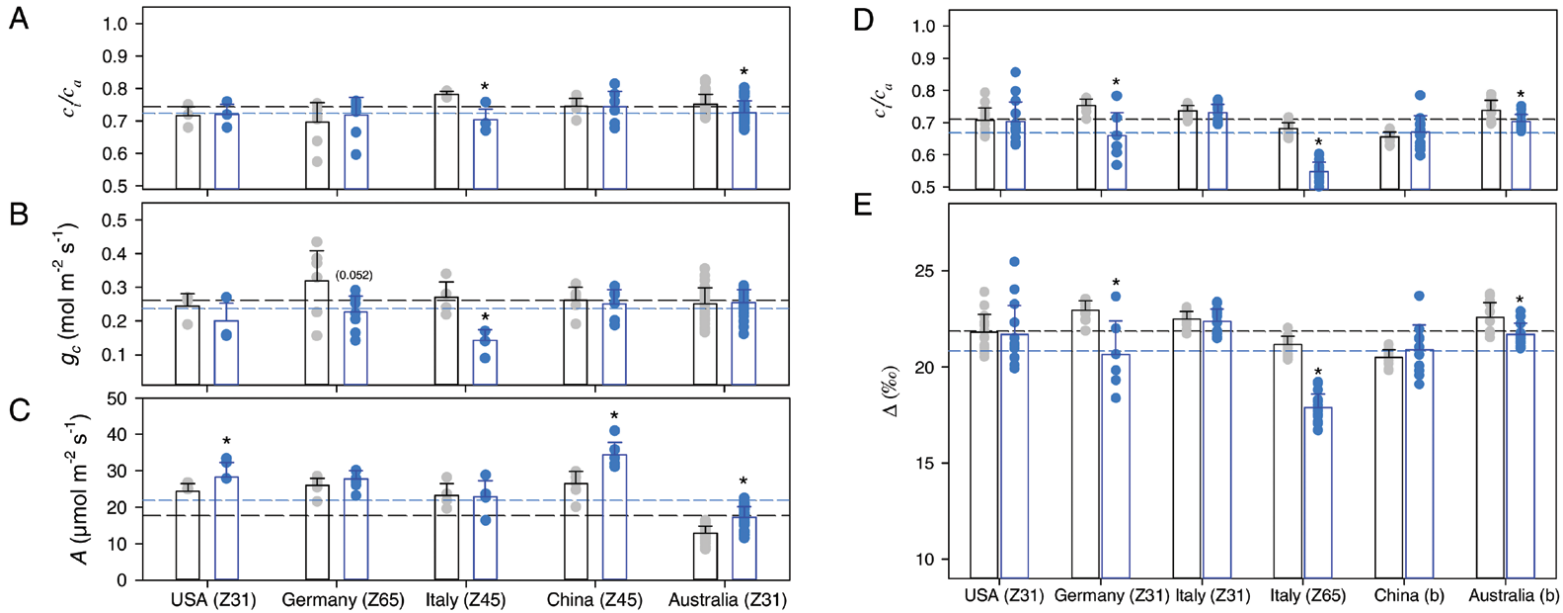
Photosynthetic parameters in wheat grown under ambient (grey) or elevated (550 µmol mol^−1^, blue) CO_2_ mole fraction. (A–C) Leaf gas exchange properties: intercellular-to-atmospheric CO_2_ ratio (*c*_i_/*c*_a_) (A), stomatal conductance for CO_2_ (B), and net photosynthesis (C). (D, E) ^12^C/^13^C carbon isotope fractionation (Δ) measured using leaf total organic matter δ ^13^C (E) and calculated average *c*_i_/*c*_a_ values (E). Asterisks indicate significant differences (*P*<0.05) between ambient and elevated CO_2_. *P*-values very close to statistical significance are given in parentheses. Dashed lines show average values across all countries. The developmental stage (BBCH) for plants in each country is given in parentheses; for China and Australia, values obtained at both BBCH stages 31 and 65 were pooled together (indicated by b). ND, not determined. (This figure is available in colour at *JXB* online.)

The photosynthetic activity of flag leaves was assessed using measurements of gas exchange at various times of the development cycle (shown as BBCH stage numbers), at the CO_2_ mole fraction (*c*_a_) used during growth (Fig. 1). Overall, there was little variation in the photosynthetic rate, which was ~22 µmol m^−2^ s^−1^ regardless of CO_2_, with the following properties (Fig. 1C): (i) in China, the USA, and Australia, there was a significantly higher photosynthetic rate at elevated CO_2_, and (ii) in Australia, plants generally had lower photosynthetic rates compared with other countries. These effects were unrelated to stomatal conductance (Fig. 1B), which was roughly constant at 0.25 mol m^−2^ s^−1^; however, CO_2_ had a significant depressing effect in plants grown in Italy. As a result, there was little variation in the intercellular-to-external CO_2_ ratio (*c*_i_/*c*_a_) (Fig. 1A), which was always ~0.75, except in Italy (*c*_i_/*c*_a_ <0.7) and in Australia (*c*_i_/*c*_a_ ≈0.7) at elevated CO_2_. The natural carbon isotope abundance (δ ^13^C) in raw leaf matter was measured, and ‘average’ *c*_i_/*c*_a_ (i.e. across the leaf lifespan) was estimated using the simplified equation of Farquhar *et al*. (1982) (Fig. 1D, E). The ^12^C/^13^C fractionation (raw matter versus source CO_2_) was within 20–22‰, showing that average *c*_i_/*c*_a_ was always ~0.7. However, the isotope fractionation was significantly lower (and therefore so was average *c*_i_/*c*_a_) in Germany, Italy, and Australia at elevated CO_2_, suggesting a long-term decrease in stomatal (or internal) conductance and/or an increase in photosynthetic capacity at elevated CO_2_. In addition, there were no significant changes either between CO_2_ conditions or among countries in ABA content (data not shown).

### Leaf metabolism

**Fig. 2.**
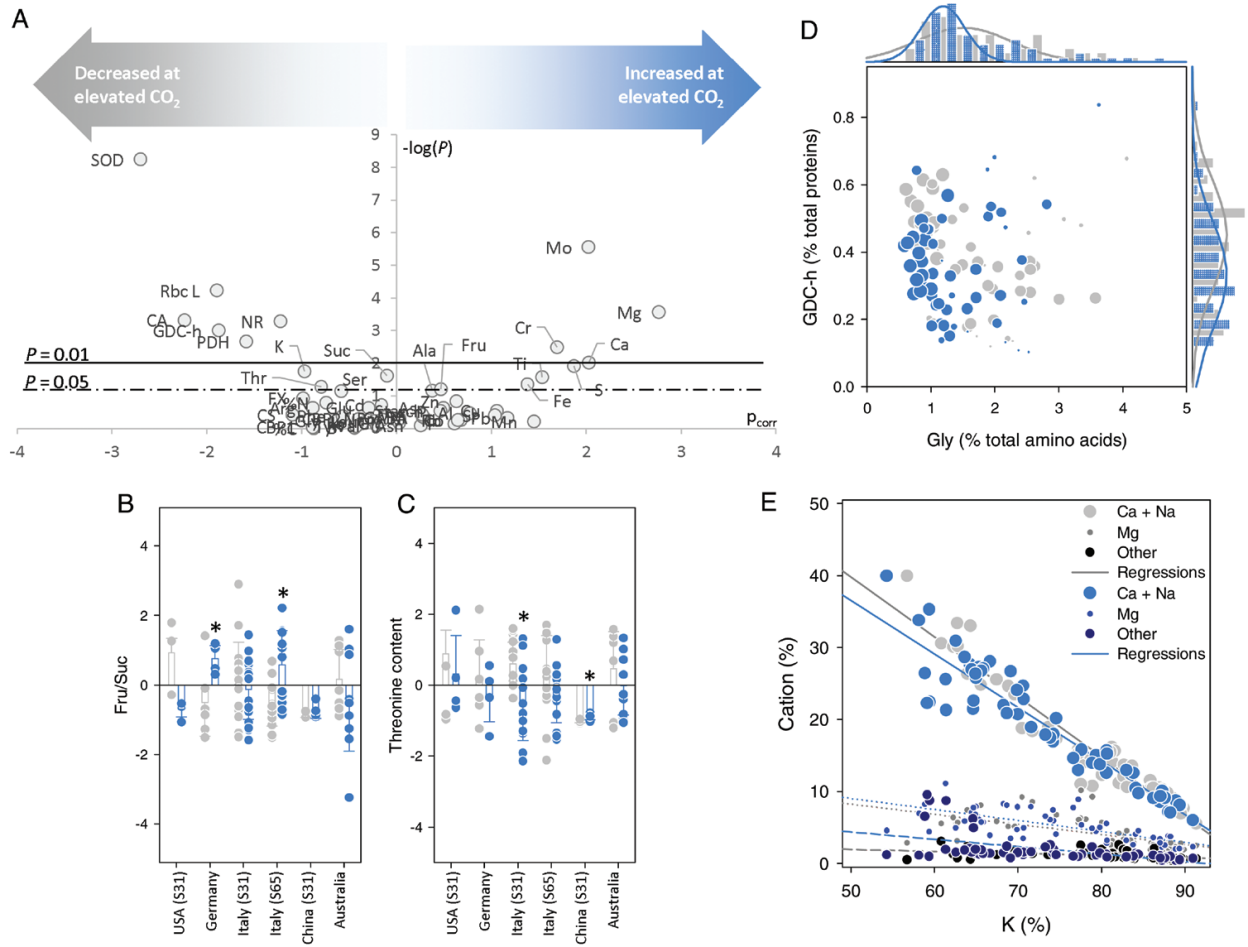
Metabolism of wheat leaves in plants grown under ambient (grey) or elevated (550 µmol mol^−1^, blue) CO_2_ mole fraction. (A) Volcano plot [–log(*P*-value) from ANOVA versus the loading p_corr_ from OPLS] showing the best discriminating components (metabolites, proteins, and elements) associated with the effect of CO_2_ enrichment. Threshold *P*-values (0.01 and 0.05) are shown with horizontal solid and dashed lines. (B, C) Relative fructose-to-sucrose ratio (B) and relative threonine content (C). In these panels, cultivars are pooled together for each country. Asterisks indicate significant differences (*P*<0.05) between the different average values. (D) Distribution of data points in the bi-plot showing the relative content of glycine decarboxylase h subunit (GDC-h) and glycine (Gly) (as a percentage of total proteins and total amino acids, respectively), with frequency plots on each axis. Countries are represented by different symbol sizes. (E) Relationship between K content and other cations under elevated and ambient CO_2_, with linear regressions (all significant, *P*<0.05). (This figure is available in colour at *JXB* online.)

The contents of amino acids, sugars, macro- and microelements, and some target proteins were analyzed in flag leaves (contents were expressed relative to dry weight). A statistical analysis combining multivariate (OPLS) and univariate (two-way ANOVA) statistics was performed and results showing the effect of CO_2_ are represented as a volcano plot in Fig. 2A. In both the OPLS and ANOVA, two factors were considered: CO_2_ and ‘conditions’ (comprising country, species/cultivar, and growth stage). Growth at elevated CO_2_ led to an increased content of several elements (Mo, Mg, Ca, Cr, Ti, Fe, and S) and a decrease in K. Several enzymes [superoxide dismutase, Rubisco (large subunit), carbonic anhydrase, pyruvate dehydrogenase, and nitrate reductase] had a lower content at elevated CO_2_, and so had threonine. There was a tendency to have more fructose and less sucrose at elevated CO_2_ (significant changes in ANOVA) but OPLS loadings were rather small, showing that the change in sugar composition was not a strong marker of growth at elevated CO_2_. When expressed as a percentage of total amino acids and proteins, glycine and glycine decarboxylase (GDH subunit h) were lower (*P*<0.001) under elevated CO_2_ (Fig. 2B), likely reflecting lower photorespiration activity. When examined separately for each country, the fructose-to-sucrose ratio appeared to be significantly different in Germany and Italy (Fig. 2C), and threonine content appeared to be significantly different in Italy and China (Fig. 2D), showing that the effect of elevated CO_2_ was country-specific (i.e. it depended on environmental local conditions). This phenomenon was clearly visible in the results of the two-way ANOVA, where several features appeared to be significant for the CO_2_ × conditions effect (Supplementary Fig. S2). For example, under elevated CO_2_, there was a notably lower content of amino acids in Australia, nearly opposite effects on starch content in the USA and Australia, and a strong decrease in the content of several microelements in China. Nevertheless, the patterns of mineral elements were the same across all countries and CO_2_ conditions. In fact, there was as expected an antagonism between major cations, with Ca + Na or Mg being negatively correlated with K (Fig. 2E). However, there was little difference in this correlation between ambient and elevated CO_2_. In other words, the significant decrease in K and increase in Ca and Mg at elevated CO_2_ (Fig. 2A) followed the same cation balance relationship as under ambient CO_2_.

### Grain metabolism

**Fig. 3.**
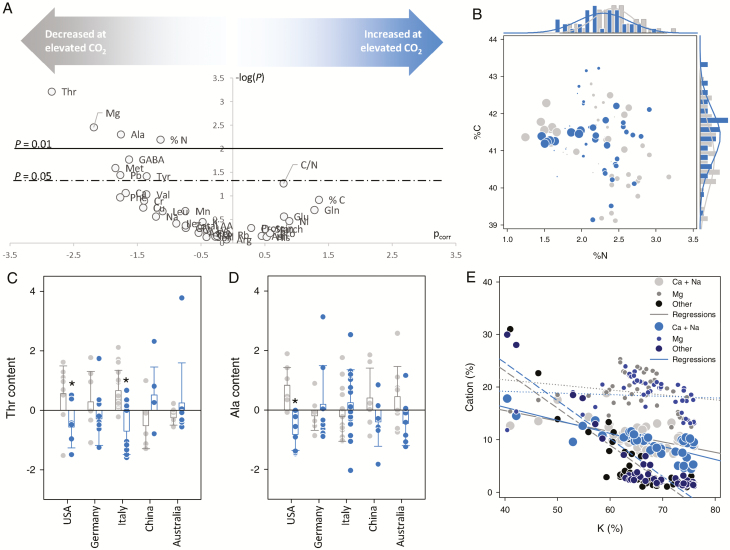
Metabolism of wheat grains in plants grown under ambient (grey) or elevated (550 µmol mol^−1^, blue) CO_2_ mole fraction. (A) Volcano plot [–log(*P*-value) from ANOVA versus the loading p_corr_ from OPLS] showing the best discriminating components (metabolites and elements) associated with the effect of CO_2_ enrichment. Threshold *P*-values (0.01 and 0.05) are shown with horizontal solid and dashed lines. (B) Distribution of data points in the bi-plot showing elemental C and N content, with frequency plots on each axis. Countries are represented by different symbol sizes. (C, D) Relative threonine (C) and alanine (D) content. In these panels, cultivars are pooled together for each country. Asterisks indicate significant differences (*P*<0.05) between control and elevated CO_2_. (E) Relationship between K content and other cations under elevated and ambient CO_2_, with linear regressions (all significant, *P*<0.05, except for Mg). (This figure is available in colour at *JXB* online.)

The contents of free amino acids, macro- and microelements, and total proteins and starch were quantified in mature grains (relative to dry weight). As in leaves, an analysis combining univariate (ANOVA) and multivariate (OPLS) statistics was performed and presented as a volcano plot (Fig. 3A). Interestingly, no component appeared to be significantly increased at elevated CO_2_. By contrast, there were significant decreases in several elements (N, Mg, and Pb), amino acids (methionine, threonine, alanine, and tyrosine), and the glutamate derivative γ-aminobutyrate (GABA). Although nitrogen content (%N) was found to be lower, there was no significant change in protein content. Such a decrease in N content could be explained by changes in the concentration of many N-containing compounds (e.g. free amino acids, polyamines, or insoluble proteins not accounted for in our assay), which in total sum up to a larger and significant change in overall %N. When plotted together, %N and carbon content (%C) showed considerable scattering across countries (Fig. 3B), with a difference greater than 1.5% in %N between samples. The effect of elevated CO_2_ on alanine and threonine appeared to be condition-specific, with significant differences between conditions found in the USA and Italy (Fig. 3C, D). More generally, the two-way ANOVA showed that amino acids, %N, and Pb were significant for the CO_2_ × conditions effect (Supplementary Fig. S3). Elevated CO_2_ did not lead to significant changes in major cations (K, Ca, Na, and Mg) (Fig. 3E). In fact, Ca + Na and Mg were negatively correlated with K, reflecting cation balance, and the correlation was not changed by elevated CO_2_; that is, the lower Mg content at elevated CO_2_ (Fig. 3A) was not associated with a change in the Mg/K balance in grains.

### Relationships with yield

**Fig. 4.**
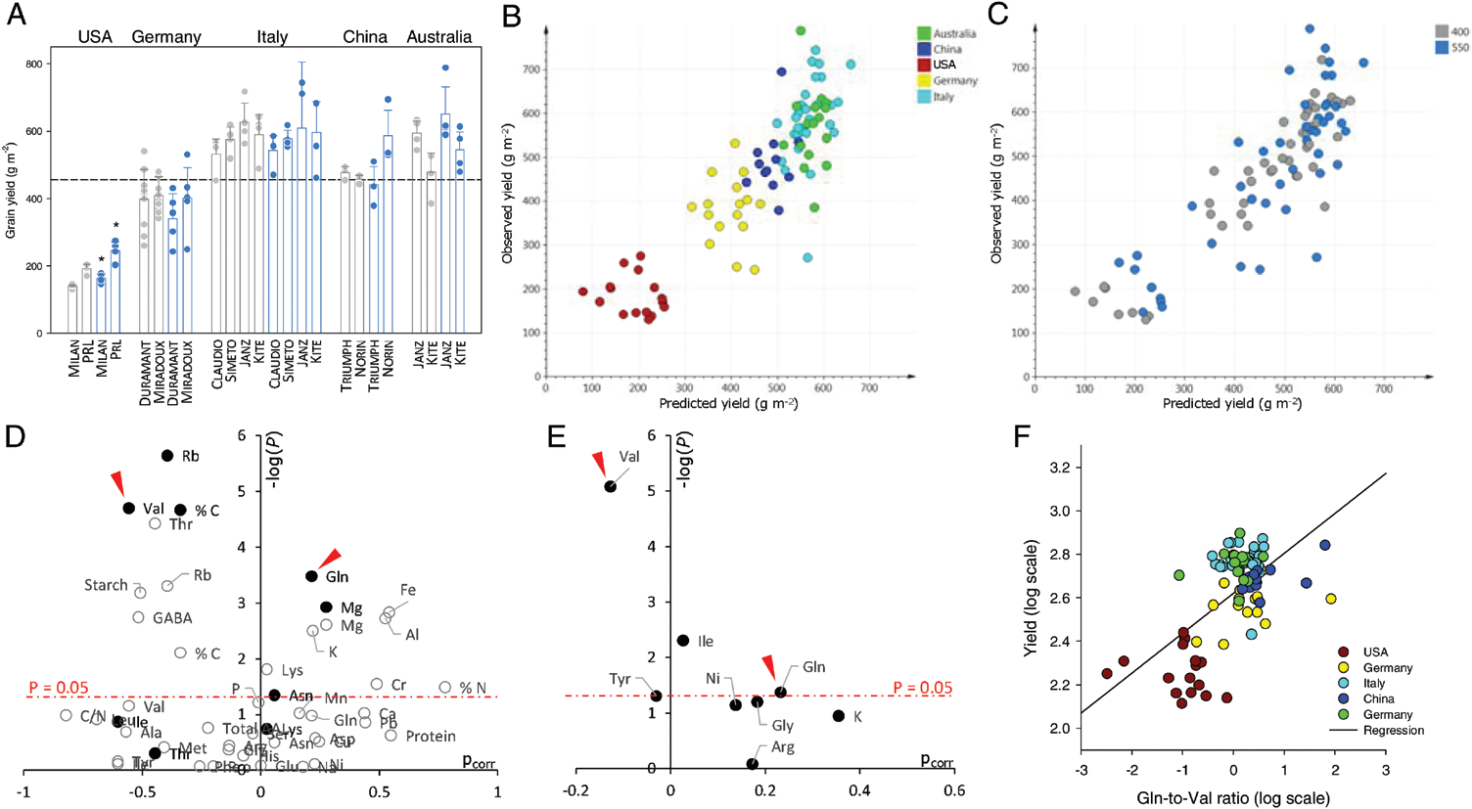
Yield analysis. (A) Yield (in g grains m^−2^) of the different cultivars and countries. Asterisks indicate significant differences (*P*<0.05) between control (grey) and elevated (blue) CO_2_. (B, C) Relationship between observed yield and yield predicted using the OPLS model (comprising country+cultivar as a qualitative X variable), differentiating countries (B) or CO_2_ levels (C) (*R*^2^=0.75). (D) Volcano plot [–log(*P*-value) versus OPLS loading p_corr_] showing the importance of variables for statistical analysis: *P*-values from separate linear regressions (each variable taken separately) (white circles), or *P*-values from variable elimination and linear model (black circles) against OPLS loadings (both univariate and OPLS models without country+cultivar as a qualitative X variable). (E) *P*-values from variable elimination and linear model against OPLS loadings (both univariate and OPLS models with country+cultivar as a qualitative X variable). In D and E, the horizontal dashed line indicates the *P*-value threshold of 0.05, and arrowheads indicate valine (Val) and glutamine (Gln). (F) Relationship between observed yield and the glutamine-to-valine ratio (log scales). The solid line represents the linear regression (*R*^2^=0.37). (This figure is available in colour at *JXB* online.)

There were considerable differences in grain yield between countries, with low values in the USA (≈200 g grain m^−2^) and high values in Australia and Italy (≈600 g grain m^−2^) (Fig. 4A). The difference in yield between countries was unsurprising and resulted from seasonal differences, as well as differences in climatic conditions, fertilization (Table 1), and soil composition (Supplementary Fig. S1), or sowing density (which ranged within 120–350 m^−2^). To gain insight into the metabolic determinants of yield, we conducted both a multivariate OPLS analysis using yield as a predicted quantitative Y variable, and a univariate analysis by linear regression. The OPLS analysis generated a good statistical model (*R*^2^=0.75) with good robustness (*Q*^2^=0.67) and very high significance (*P*_CV-ANOVA_=10^–16^). When observed and predicted yield were plotted together, it was clear that the relationship was driven partly by differences between countries (Fig. 4B) and not at all by the CO_2_ treatment (Fig. 4C). Accordingly, there were no significant CO_2_ effects, except in the USA (Fig. 4A). The best drivers of yield were investigated using volcano plots. Two volcano plots are shown here: the first (Fig. 4D) is associated with an analysis that disregards ‘conditions’ (countries and species/cultivars), while the second (Fig. 4E) incorporates ‘conditions’ as a variable. In Fig. 4D, *P*-values are associated with a variable-by-variable linear regression or with a multiple linear regression including variable elimination (where variables that are not best correlated with yield are discarded) (Supplementary Fig. S4). Regardless of country and cultivar, the yield appeared to be significantly and positively related to micro- and macroelements (Fe, Al, K, Mg, Cr, and N) and negatively related to starch, threonine, Rb, and %C. After variable elimination upon multiple linear regression, only six features were significantly related to yield: Mg, glutamine, and asparagine (positively related), and Rb, valine, and %C (negatively related). Among these features, some appeared to be significant because of the confounding factor of country of origin (Fig. 4B, C). When this effect was removed by incorporating ‘conditions’ as a variable, the two most significant drivers were glutamine (increased) and valine (decreased) (Fig. 4E). Tyrosine and isoleucine were found to be significant (*P*<0.05) but their loading value was close to zero, showing that their impact (in multivariate analysis) was numerically very small. When plotted separately, there was a positive relationship between yield and the glutamine-to-valine ratio in grains (Fig. 4F).

**Table 1.** Growth conditions associated with the CO_2_ enrichment experiments in this study

Site general information					Climatic conditions during growth				Fertilization	
Country	Location	Cultivation time window	Species and cultivars	Sowing month	P_tot_ (mm)	T_av min_ (°C)	T_av max_ (°C)	Cumulated d∙°C	Nitrogen (kg N ha^−1^)	Elemental N-P_2_O_5_-K_2_O
USA	Beltsville	16/10/15–7/6/16	*T. durum* Milan, PRL	October	141	4.2	25.9	981	250	10-10-10
Italy	Fiorenzuola	9/11/15-11/7/16	*T. aestivum* Janz, Kite; *T. durum* Claudio, Simeto	November	347	10.3	27.8	1774	183	15-15-15
Germany	Hohenheim	5/4/16-1/8/16	*T. durum* Miradoux, Duramant	April	271	2.6	24.8	1679	202	10-5-5
China	Beijing	6/10/15–20/6/16	*T. durum* Norin, Triumph	October	307	10.9	30.8	1843	200	20-16-9
Australia	Horsham	1/06/16–21/12/16	*T. aestivum* Janz, Kite	June	334	9.0	23.1	2394	50	10-1-10

Soil mineral conditions are further documented in Supplementary Fig. S1. Cumulated d∙°C, cumulated temperature during cultivation period (in days∙°C); P_tot_, total precipitation; T_av min_, average minimum daily temperature; T_av max_, average maximum daily temperature.

## Discussion

Here, we used five FACE sites in different countries, with different wheat cultivars/species, to investigate the metabolic effects of elevated CO_2_ in leaves and grains. Our results show that elevated CO_2_ (i) had a limited effect on photosynthesis rate and grain yield, reflecting photosynthetic acclimation; (ii) caused a decline in proteins involved in photosynthesis, photorespiration, or N assimilation in leaves; and (iii) altered grain quality, with lower contents of amino acids and mineral nutrients. In addition, a quantitative analysis of yield suggested that high yield values correlate with higher glutamine, K, and Mg contents and lower valine content.

### Growth at elevated CO_2_ had a limited impact on crop yield

Crop yield and photosynthetic responsiveness to elevated CO_2_ depend considerably on surrounding (local) environmental conditions. Here, we found that with the exception of the FACE site in the USA, grain yield was not significantly affected by elevated CO_2_. In practice, changes in wheat yield in response to high CO_2_ are determined by the cultivar(s), fertilization protocols, and interactions with other environmental factors such as water availability and temperature. The absence of an effect of CO_2_ was probably linked to the lack of significant changes in assimilation (mechanisms are further discussed below). Accordingly, several previous studies have shown that the initial stimulation by CO_2_ can be compensated for by acclimation (reviewed in Ainsworth and Rogers, 2007). The meta-analyses carried out by Galmés *et al*. (2013) showed that under elevated CO_2_ conditions, Rubisco content is the primary driver in the regulation of Rubisco activity and, consequently, photosynthetic activity. Within this context, the absence of an effect of elevated CO_2_ on crop yield could be linked to limitations in N assimilation and altered leaf C sink/source balance (Ainsworth and Rogers, 2007). Photosynthetic performance is believed to be affected by two key factors: the CO_2_ concentration in the chloroplast (*c*_c_) and the carboxylation capacity, which is linked to leaf N content. Here, the absence of significant differences in stomatal conductance (accompanied by the lack of an effect on leaf ABA content) suggests that stomatal limitation of CO_2_ diffusion was a minor component of acclimation under our conditions. The lower content of nitrate reductase (and other proteins) suggests an inhibitory effect of elevated CO_2_ on N metabolism, as previously observed in wheat leaves (Jauregui *et al.*, 2015). The inhibition of nitrate assimilation under elevated CO_2_ in wheat has been suggested to reflect lower electron allocation to nitrite reductase in the chloroplast and/or the inhibition of the GS/GOGAT cycle driven by photorespiration (Bloom *et al.*, 2014). Here, the lower contents of ferredoxin, chlorophyll binding proteins, and glycine decarboxylase under elevated CO_2_ would be consistent with a down-regulation of N assimilation. 

Besides the effect of CO_2_ (or lack thereof), our study shows important differences in yield between locations and cultivars. The lowest values were found in the USA, where sowing density (120 m^−2^), precipitation, and cumulated day·°C values were low (Table 1), and seedling mortality was pronounced in the year of the FACE experiment due to bad weather. The high yield values observed in China were probably explained by higher water availability. We note that the FACE site in the USA, where elevated CO_2_ had a significant effect on yield, had the highest N fertilization rate (250 kg ha^−1^) despite low plant sowing density and low cumulated days∙°C; this suggests that perhaps the two most important factors for CO_2_ responsiveness were available N and intercepted light (minimal shading) under our conditions. Differences between locations could also have originated from the contrasting behaviour of the two species of wheat studied, with bread wheat exhibiting generally higher yield values than durum wheat. This species effect comes from the fact that durum wheat is more water conservative and thus more suitable to grow in stressful environments, while bread wheat is believed to have a higher yield potential (Slafer *et al.*, 1996; Marti and Slafer, 2014).

### Photosynthetic effects of elevated CO_2_

Growth at elevated CO_2_ generally leads to a down-regulation of some photosynthetic parameters, such as carboxylation efficiency, maximal carboxylation velocity, and CO_2_ conductance (see Introduction). Here, photosynthesis increased by a modest but significant amount at elevated CO_2_ (in the USA, China, and Australia) or did not increase at all (in Italy and Germany). In Italy, the lack of a stimulating effect of CO_2_ mole fraction was associated with a decrease in stomatal conductance (Fig. 1B). It is also possible that internal conductance decreased under elevated CO_2_, and this could contribute to explaining why the ^12^C/^13^C isotope fractionation did not increase much (with apparent *c*_i_/*c*_a_ staying at ~0.7 despite the increase in CO_2_ mole fraction) (Fig. 1E). In addition, there was probably a decrease in carboxylation capacity, as suggested by the significantly lower Rubisco content in leaves (Fig. 2), and *A*/*c*_i_ response curves for wheat grown in Germany suggest a lower *V*_cmax_ (non-significant) and *J*_max_ (*P*<0.05) (Supplementary Fig. S5). More generally, elevated CO_2_ led to a down-regulation of the photosynthetic and photorespiratory machinery, with less carbonic anhydrase, glycine decarboxylase, and superoxide dismutase, an enzyme of redox metabolism (as reported previously in Aranjuelo *et al.*, 2015). As a result, the lower content of major enzymes such as Rubisco was accompanied by a decline in %N in some countries (the USA, China, and Australia) but not others. The origin of this effect of elevated CO_2_ could be lower biosynthesis of proteins or an increase in protein degradation, for example, earlier remobilization of proteins to facilitate export of N from leaves to developing grains (Sicher and Bunce, 1998; Zhu *et al.*, 2009). Earlier remobilization seems unlikely, since leaves were sampled at stage Z31 (stem elongation with first node above tillering node) or Z65 (anthesis, 50% of anthers mature), that is, before the onset of remobilization. Therefore, it is probable that elevated CO_2_ down-regulated N metabolism, causing a general decrease in the biosynthesis of major proteins and thus potentially photosynthesis. Despite the change in Rubisco content, the net effect on photosynthesis also depends on other enzyme activities, because net assimilation is not highly sensitive to Rubisco content. For example, other enzymes, such as Rubisco activase, are crucial for carboxylation efficiency. We have previously shown using proteomics that elevated CO_2_ leads to a decline in Rubisco activase content (Aranjuelo *et al.*, 2015). Here, our analysis focused on a number of specific proteins, which did not include Rubisco activase. Photosynthetic products (sugars) were affected by CO_2_ mole fraction, with significantly less sucrose and generally more fructose (*P*=0.06); as a result, the fructose-to-sucrose ratio was larger at elevated CO_2_ in two countries (Germany and Italy) (Fig. 2B). In other words, elevated CO_2_ was associated with a reconfiguration of sugar metabolism, likely including a larger allocation of carbon to starch and a futile sucrose synthesis–degradation cycle.

### Effect of CO_2_ on grain composition

We found that elevated CO_2_ had a clear effect on grain composition, leading to lower contents of N, amino acids, and microelements (Fig. 3). The general effect of elevated CO_2_ on N content (and proteins) in grains has been documented before, and has been suggested to be linked to perturbations in both leaf-to-grain N transport (e.g. glutamine) and amino acid metabolism in grains (e.g. in the lysine degradation pathway; see the Introduction and Soba *et al.*, 2019). Here, it is remarkable that Mg was found to be more abundant in leaves but less abundant in grains, suggesting that elevated CO_2_ compromised microelement remobilization (the same occurred with Ca but this was not significant in grains). Nutrient quantitation during grain filling in different cultivars of spring wheat has suggested that Mg is remobilized from the lower stem and leaves to grains, with little effect of cultivar (Tiryakioglu *et al.*, 2014). Growth at elevated CO_2_ has been found to affect Mg content in wheat (Sanchez de la Puente *et al.*, 2000; Högy *et al.*, 2013; Aranjuelo *et al.*, 2015; Beleggia *et al.*, 2018). Mg remobilization involves chlorophylls and Rubisco degradation in leaves, and Mg circulation via the phloem. This raises the question of whether phloem transport was affected by elevated CO_2_ in the current study. On the one hand, glutamine and glutamate were found to be more abundant in grains at elevated CO_2_ (Fig. 3), suggesting that phloem N export from leaves was not affected. On the other hand, leaves contained significantly less K, which is the cornerstone of phloem ion movement. Therefore, our data do not provide a firm answer to this question. Nevertheless, we recognize that the higher glutamine (glutamate) content in grains could also have originated from a lower efficiency of glutamine utilization and thus a lower incorporation of N to anabolism. Grains were specifically depleted in GABA, methionine, tyrosine, alanine, and threonine. Interestingly, these amino acids belong to distinct pathways: aspartate metabolism (methionine and threonine), glutamate degradation/GABA shunt (GABA and alanine), and aromatics (tyrosine). The common branching point is pyruvate, since aspartate can come from anaplerotic phospho*enol*pyruvate (PEP) carboxylase fixation (which forms oxaloacetate), alanine comes from pyruvate, and the biosynthesis of aromatics requires PEP. Therefore, it is possible that the decrease in amino acids was caused by a specific effect of elevated CO_2_ on (phospho*enol*)pyruvate synthesis via glycolysis. For example, pyruvate Pi dikinase, which resynthesizes PEP from pyruvate, has been shown to be essential for starch accumulation at the late grain-filling stage (Prioul *et al.*, 2012).

Importantly, these metabolic effects depended on ‘conditions’, that is, country and species/cultivar (Supplementary Figs S2, S3). It was not possible here to separate the specific contributions of country and species/cultivar, since not all plant lines were cultivated in each country. There was nevertheless a strong effect of location, with the effect of elevated CO_2_ on some micro- and macro-elements being country specific. For example, the effect of elevated CO_2_ on grain Pb content was largest in Australia, where Pb was the least abundant at the field location (Supplementary Fig. S1). Similarly, there was a very limited effect of CO_2_ on grain N content (with generally low values) in Australia (Supplementary Fig. S3), where N fertilization was the lowest (Table 1), while the effect on leaf N content was strong (Supplementary Fig. S2). There was also a strong effect of CO_2_ on grain free amino acid content in both Australia and the USA, despite the fact that the sites in the two countries used different fertilization levels and wheat species. Therefore, our results show the importance of growth conditions (i.e. location) to delineate the effects of elevated CO_2_; in particular, soil composition (and not only N fertilization) seems to be important. Yet, the decrease in threonine and Mg content in grains was independent of ‘conditions’, that is, it was not associated with a CO_2_ × conditions interaction effect.

### Potential metabolic markers of yield

We used the dataset to examine possible relationships between yield and grain metabolic properties, using multivariate and univariate statistics (Fig. 4). Because the country of origin (and associated conditions such as climate, fertilization, and sowing density) and species/cultivar (i.e. ‘conditions’) represented a confounding factor, we carried out two types of analyses: (i) independent of and (ii) accounting for ‘conditions’. In doing so, we assumed that the variable ‘conditions’ can be a driver of yield regardless of other variables (e.g. contents of metabolites or micro-/macro-elements). Growth at elevated CO_2_ was not a confounding factor, since there was no significant effect of CO_2_ on yield overall (Fig. 4A, C). Only two significant and strong drivers of yield remained after the elimination of the ‘conditions’ effect: glutamine and valine (Fig. 4E, F).

This result highlights the well-known role of glutamine in grain protein synthesis: glutamate is a major amino acid in wheat stem phloem (Hayashi and Chino, 1986) used as a primary source of N for conversion to glutamine and, furthermore, glutamine is the most represented residue in accumulated proteins. It has been shown that glutamine accounts for ~40% of free amino acids not only in phloem sap from the spikelet peduncle but also in endosperm cavity sap, showing its crucial role as a N source during grain development (Fisher and Macnicol, 1986). In addition, glutamine has the highest supply rate from the vascular bundle to the developing endosperm, at ~0.5 µmol grain^−1^ d^−1^ (Ugalde and Jenner, 1990).

Valine was negatively related to yield (Fig. 4E), suggesting that increased valine degradation rather than valine synthesis is beneficial to grain production. Proteomics analyses have shown that acetolactate synthase, which catalyses the first step of valine and leucine biosynthesis, is most abundant at the beginning of grain development (pre-filling stage) and then declines up to maturity (Tasleem-Tahir *et al.*, 2012), while methylmalonate semialdehyde dehydrogenase, which is involved in branched-chain amino acid degradation, appears at the last stage of grain filling (Vensel *et al.*, 2005) and its content increases with ABA (Zhang *et al.*, 2012). Accordingly, metabolic profiling has shown that branched-chain amino acids (valine, leucine and isoleucine) tend to increase during grain filling and then sharply decline (Shewry *et al.*, 2012). Valine metabolism can have several roles. First, valine degradation generates acetyl-CoA (via methylmalonate semialdehyde), which can in turn be used for respiration or lipid synthesis. Second, valine can be converted to leucine via 2-oxoisovalerate. Labelling with ^14^C-valine in developing wheat spikes has shown up to 30% conversion to leucine (Kolderup, 1978), and during grain development, the valine content correlates with that of leucine (Martín Del Molino *et al.*, 1988). Interestingly, a quantitative trait locus analysis has shown that 2-methylmaleate, an intermediate of leucine synthesis, correlates positively with the number of tillers and thermal time to heading, and negatively with yield (Hill *et al.*, 2015).

## Conclusions

Taken as a whole, our results show that growth at elevated CO_2_ in FACE experiments had different effects on leaf and grain metabolism, but no significant effect on yield. Elevated CO_2_ appeared to be detrimental to photosynthesis and leaf proteins (e.g. Rubisco large subunit) and to alter grain composition, in particular %N, amino acids (e.g. threonine and alanine), and minerals (Mg). The yield was related to metabolic features of grains but this relationship was not influenced by elevated CO_2_.

The lack of a positive effect of CO_2_ fertilization can be explained not only by the down-regulation of carbon fixation (i.e. of several photosynthetic parameters) but also by an effect on grain metabolism itself. In particular, elevated CO_2_ appeared to be detrimental to N metabolism, with a decrease in several amino acids and, accordingly, a positive effect of elevated CO_2_ on yield was observed only in the USA, where N fertilization was the highest.

Elevated CO_2_ also had an effect on Mg redistribution between source leaves and grains. The detrimental effect of CO_2_ on the content of microelements has been found elsewhere for Zn (Myers *et al.*, 2014). A meta-analysis has recently highlighted the decrease in the content of many elements (including macroelements such as S) in crops cultivated at elevated CO_2_ (Loladze, 2014). Interestingly, recent experiments in which Mg availability was varied have shown that low Mg causes a significant decline in grain starch content and yield (Ceylan *et al.*, 2016). Here, despite significantly lower Mg content in grains at elevated CO_2_, starch content and yield were not significantly affected, suggesting that the decrease in Mg associated with elevated CO_2_ was too small (<10%) to affect yield. In addition, the relationship between Mg content in grains and yield is not driven by genotype (Oury *et al.*, 2006). We nevertheless recognize that further work is required to determine the reason for the decreased Mg content in grains found here. For example, isotopic labelling with ^25^Mg (or ^26^Mg) would be helpful to examine the role played by phloem Mg transport during remobilization from leaves at elevated CO_2_.

The overall effect on N, K, and Mg nutrition found here raises the question of whether specific fertilization management strategies could compensate for the lack of effect of elevated CO_2_ observed at most of the FACE sites. Previous studies compared ammonium-based and nitrate-based fertilizers and found that with nitrate, plants tended to show higher photosynthetic acclimation (Bowler and Press, 1996; Geiger *et al.*, 1999; Cruz *et al.*, 2014), which in turn limits the response of production or yield to elevated CO_2_. In addition, high nitrate availability tends to exaggerate photosynthetic acclimation, due to down-regulation of the expression of photosynthetic genes (Vicente *et al.*, 2017). Therefore, in an effort to find optimal fertilization strategies adapted to local field conditions, both the quantity and the quality of N fertilizer seems to be important. The fact that nutrients other than N are also affected by elevated CO_2_ further indicates that the nutrient balance itself is also of importance. A specific multifactorial experiment (nutrient compositions × CO_2_) would be necessary to determine the best solutions to improve yield responsiveness to CO_2_. In addition, the selection of varieties with a high harvest index, which is associated with optimal allocation and efficient nutrient remobilization capacity, might be desirable to obtain better grain yield and quality at elevated CO_2_.

Finally, we found that wheat grain yield was related to glutamine and valine grain content, regardless of country and cultivar, showing the importance of amino acid metabolism for grain maturation. However, while the role of glutamine is clear (as a N source and utilization for storage proteins, which are glutamine rich), understanding the metabolic role(s) of valine requires further work, such as tracing with ^13^C-valine and isotope-assisted metabolomics. This will be addressed in a future study.

## Supplementary data

The following supplementary data are available at *JXB* online.

Fig. S1. Elemental composition of soil (top layer) at the different sites used in this study.

Fig. S2. Leaf features significant for a CO_2_ × conditions (country, cultivar, and time) effect. 

Fig. S3. Grain features significant for a CO_2_ × conditions (country, cultivar, and time) effect. 

Fig. S4. Correlation plot showing the best variables obtained by subset sampling in linear models of yield.

Fig. S5. Photosynthetic response curve for wheat cultivated in Germany.

eraa330_suppl_Supplementary_Figures-S1-S5
