# Supplementary material for: Elevated CO2 has concurrent effects on leaf and grain metabolism but minimal effects on yield in wheat
Source: J Exp Bot. 2020 Jul 20;71(19):5990–6003. doi: 10.1093/jxb/eraa330 (PMC7751139; doi:10.1093/jxb/eraa330)
Supplement: eraa330_suppl_Supplementary_Figures-S1-S5 [file eraa330_suppl_supplementary_figures-s1-s5.pdf]

Supplementary material

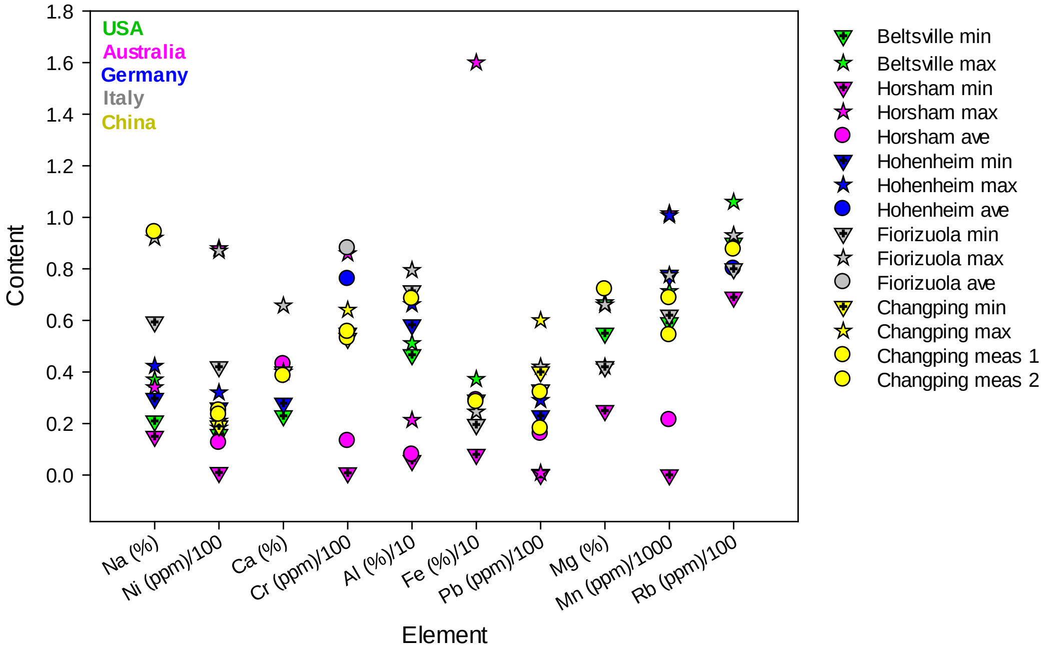

Fig. S1. Elemental composition of soil (top layer) at the different sites used in this study. For each country, maximum and minimum (and when known, average) content reported in soil composition maps are shown (data compiled from (Sultan, 2007; Smith *et al.*, 2011), Geoscience Australia (www.ga.gov.au), EuroGeoSurvey (www.eurogeosurveys.org) and Foregs Geochemical Atlas (www.gtk.fi)) as well as published values (referred to as "meas") in Changping, China (Zhang, 2016). Note the particularly variable content in iron in the Horsham region (Australia), while other nutrients are generally present at low amount. Direct measurements (made using the $CaCl_2$ extraction method of the Association of German Agricultural and Analytic and Research Institutes, VDLUFA) during the experiment in Hohenheim (Germany) indicated a free Mg content comprised between 11 and 16 mg 100 g^{-1} (0.011-0.016%).

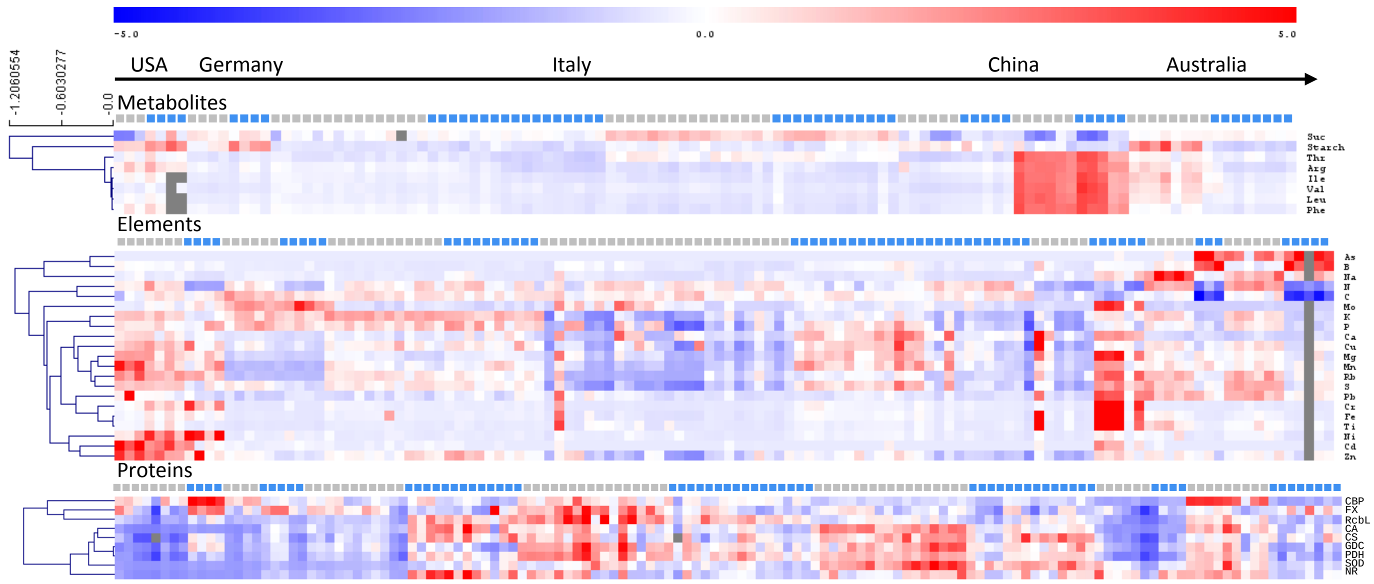

Fig. S2. Leaf features significant for a CO_2 × conditions (country, cultivar and time) effect. Data are represented as heatmaps with metabolites (upper panel), mineral elements (middle) and proteins (bottom). Samples associated with control and CO_2-enrichment are shown by grey and blue squares, respectively, on top of each heatmap. In particular, note *(i)* the lower content in several amino acids in Australia at high CO_2, and the contrasted effect of elevated CO_2 on starch content in USA and Germany, and Australia; *(ii)*, the particularly high depressing effect of elevated CO_2 on elements in China; and *(iii)*, the strong effect of elevated CO_2 on several leaf proteins (including Rubisco) in most countries except in USA. In this figure, contrary to Fig. 2 where a common set of samples was used (i.e. where all metabolites, proteins and elements were measured simultaneously), there are 3 separate heatmaps (three ANOVAs carried out separately on metabolites, elements and proteins) due to differences in sample sets.

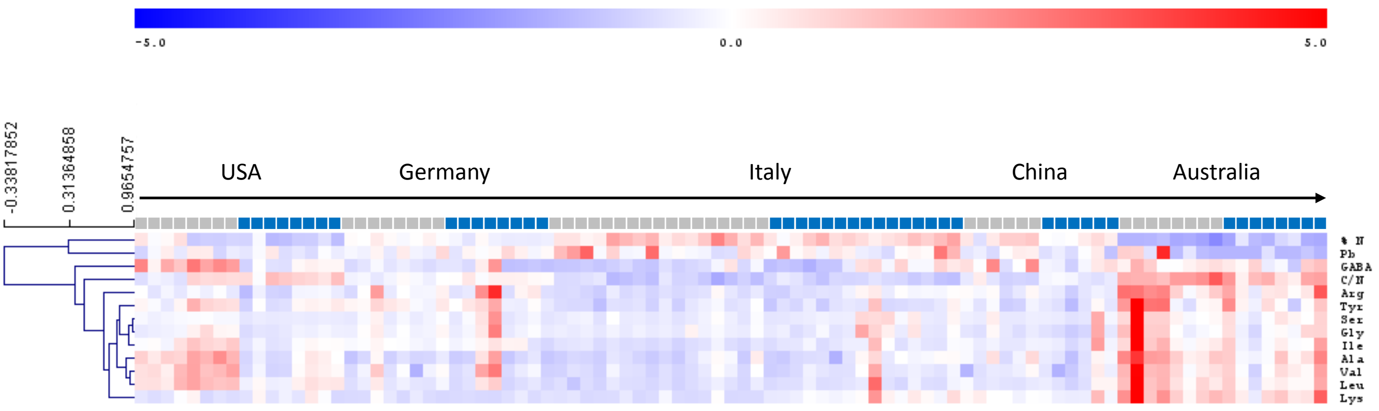

Fig. S3. Grain features significant for a CO_2 × conditions (country, cultivar and time) effect. Data are represented as heatmaps including metabolites and mineral elements. Samples associated with control and CO_2-enrichment are shown by grey and blue squares, respectively, on top of the heatmap. Note the clear depressing effect of CO_2 on amino acids in USA and Australia, and the lack thereof in Germany, Italy of China.

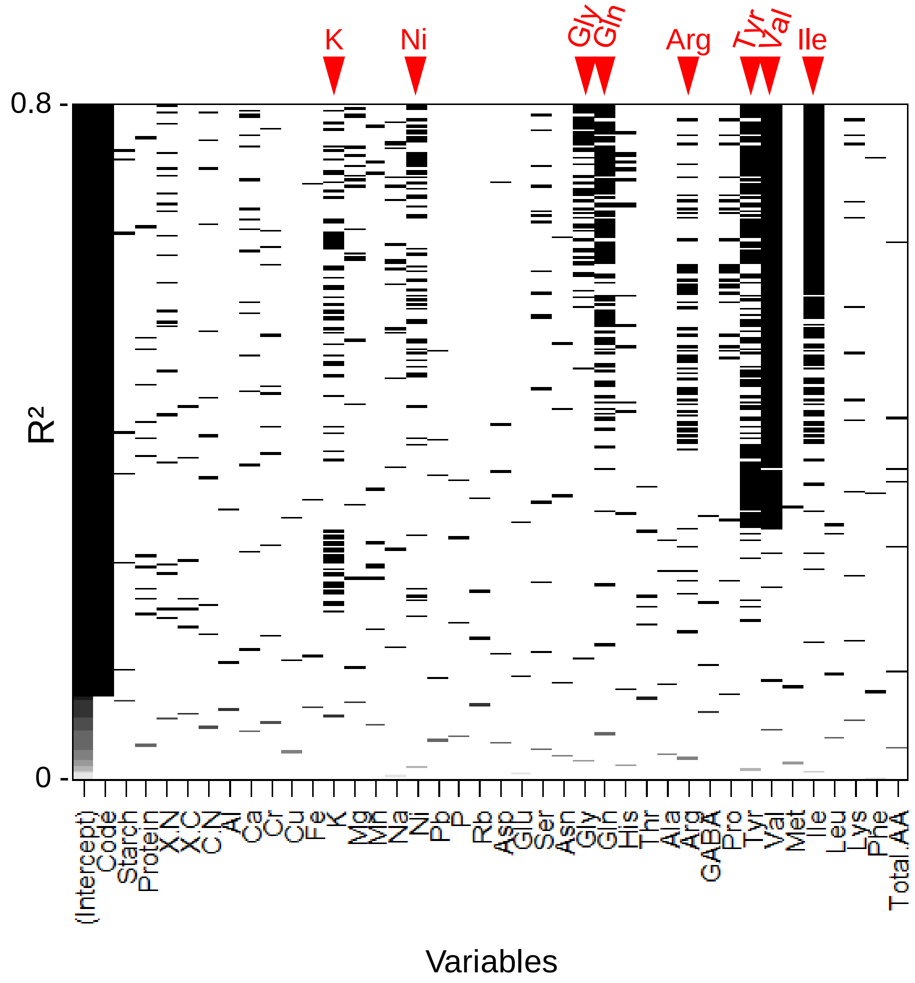

Fig. S4. Correlation plot showing the best variables obtained by subset sampling in linear models of yield. Best variables appear to be potassium (K), nickel (Ni), glycine, glutamine, arginine, tyrosine, valine and isoleucine (labelled in red on top). However, not all of them were associated with a *P*-value < 0.05 (significance of the regression; see Fig. 4e in main text). On the x-axis, "code" refer to country, and X.C (resp. X.N) refers to %C (resp. %N).

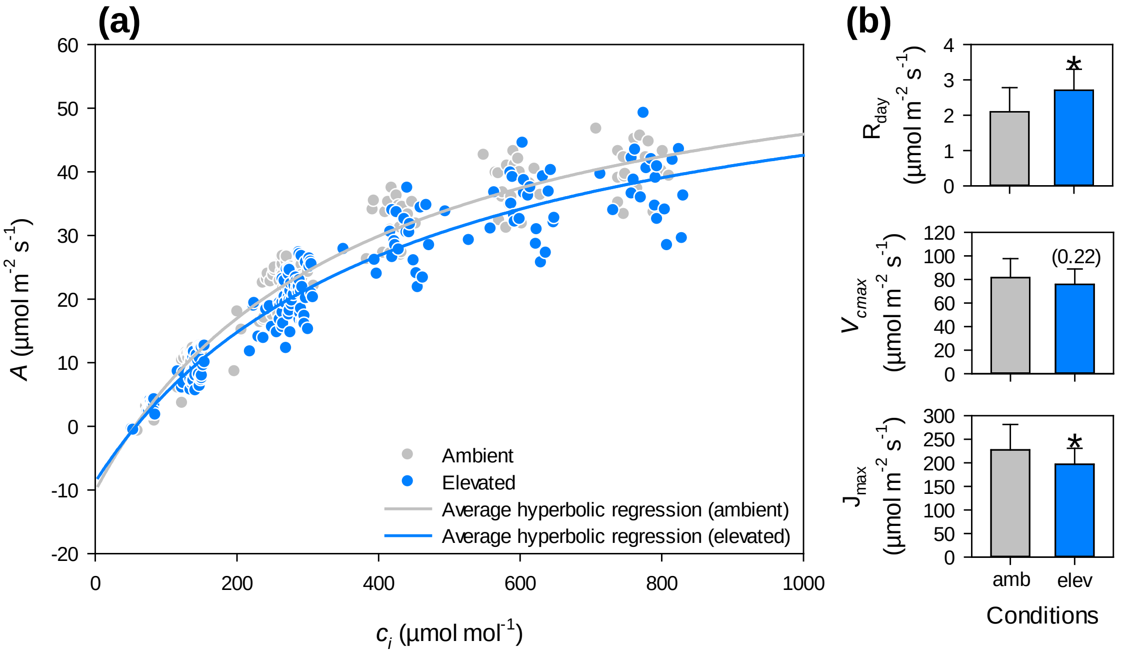

Fig. S5. Photosynthetic response curve in wheat cultivated in Germany. (a), all data plotted as a A/c_i curve with an hyperbolic regression, which generated apparent V_{cmax} estimates of 68.9 (ambient) and 66.6 (elevated) µmol m^{-2} s^{-1}. (b), mean±SD of parameters computed from each individual A/c_i curve. Asterisks stand for statistical significance ($P < 0.05$). Data shown were collected from 67 to 82 DAS.
